# Age- and sex-specific effects of a long-term lifestyle intervention on body weight and cardiometabolic health markers in adults with prediabetes: results from the diabetes prevention study PREVIEW

**DOI:** 10.1007/s00125-022-05716-3

**Published:** 2022-05-25

**Authors:** Ruixin Zhu, Ionut Craciun, Jan Bernhards-Werge, Elli Jalo, Sally D. Poppitt, Marta P. Silvestre, Maija Huttunen-Lenz, Melitta A. McNarry, Gareth Stratton, Svetoslav Handjiev, Teodora Handjieva-Darlenska, Santiago Navas-Carretero, Jouko Sundvall, Tanja C. Adam, Mathijs Drummen, Elizabeth J. Simpson, Ian A. Macdonald, Jennie Brand-Miller, Roslyn Muirhead, Tony Lam, Pia S. Vestentoft, Kristine Færch, J. Alfredo Martinez, Mikael Fogelholm, Anne Raben

**Affiliations:** 1 Department of Nutrition, Exercise and Sports, Faculty of Science, University of Copenhagen, Copenhagen, Denmark; 2 Department of Food and Nutrition, University of Helsinki, Helsinki, Finland; 3 Human Nutrition Unit, School of Biological Sciences, Department of Medicine, University of Auckland, Auckland, New Zealand; 4 CINTESIS, NOVA Medical School (NMS), Universidade Nova de Lisboa, Lisboa, Portugal; 5 Institute for Nursing Science, University of Education Schwäbisch Gmünd, Schwäbisch Gmünd, Germany; 6 Applied Sports, Technology, Exercise and Medicine (A-STEM) Research Centre, Swansea University, Swansea, UK; 7 Department of Pharmacology and Toxicology, Medical University of Sofia, Sofia, Bulgaria; 8 Centre for Nutrition Research, University of Navarra, Pamplona, Spain; 9 Centro de Investigacion Biomedica en Red Area de Fisiologia de la Obesidad y la Nutricion (CIBEROBN), Instituto de Salud Carlos III (ISCII), Madrid, Spain; 10 IdisNA Instituto for Health Research, Pamplona, Spain; 11 Finnish Institute for Health and Welfare, Helsinki, Finland; 12 Department of Nutrition and Movement Sciences, NUTRIM, School of Nutrition and Translational Research in Metabolism, Maastricht University, Maastricht, the Netherlands; 13 MRC/ARUK Centre for Musculoskeletal Ageing Research, National Institute for Health Research (NIHR) Nottingham Biomedical Research Centre, School of Life Sciences, University of Nottingham, Nottingham, UK; 14 School of Life and Environmental Sciences and Charles Perkins Centre, University of Sydney, Sydney, Australia; 15 NetUnion, Lausanne, Switzerland; 16 Clinical Research, Copenhagen University Hospital – Steno Diabetes Center Copenhagen, Herlev, Denmark; 17 Department of Biomedical Sciences, University of Copenhagen, Copenhagen, Denmark; 18 Department of Nutrition and Physiology, University of Navarra, Pamplona, Spain; 19 Precision Nutrition and Cardiometabolic Health Program, IMDEA-Food Institute, Madrid Institute for Advanced Studies, CEI UAM + CSIC, Madrid, Spain

**Keywords:** Cardiovascular disease, Men, Middle-aged people, Obesity, Older people, Weight loss, Weight loss maintenance, Women, Young people

## Abstract

**Aims/hypothesis:**

Lifestyle interventions are the first-line treatment option for body weight and cardiometabolic health management. However, whether age groups or women and men respond differently to lifestyle interventions is under debate. We aimed to examine age- and sex-specific effects of a low-energy diet (LED) followed by a long-term lifestyle intervention on body weight, body composition and cardiometabolic health markers in adults with prediabetes (i.e. impaired fasting glucose and/or impaired glucose tolerance).

**Methods:**

This observational study used longitudinal data from 2223 overweight participants with prediabetes in the multicentre diabetes prevention study PREVIEW. The participants underwent a LED-induced rapid weight loss (WL) period followed by a 3 year lifestyle-based weight maintenance (WM) intervention. Changes in outcomes of interest in prespecified age (younger: 25–45 years; middle-aged: 46–54 years; older: 55–70 years) or sex (women and men) groups were compared.

**Results:**

In total, 783 younger, 319 middle-aged and 1121 older adults and 1503 women and 720 men were included in the analysis. In the available case and complete case analyses, multivariable-adjusted linear mixed models showed that younger and older adults had similar weight loss after the LED, whereas older adults had greater sustained weight loss after the WM intervention (adjusted difference for older vs younger adults −1.25% [95% CI −1.92, −0.58], *p*<0.001). After the WM intervention, older adults lost more fat-free mass and bone mass and had smaller improvements in 2 h plasma glucose (adjusted difference for older vs younger adults 0.65 mmol/l [95% CI 0.50, 0.80], *p*<0.001) and systolic blood pressure (adjusted difference for older vs younger adults 2.57 mmHg [95% CI 1.37, 3.77], *p*<0.001) than younger adults. Older adults had smaller decreases in fasting and 2 h glucose, HbA_1c_ and systolic blood pressure after the WM intervention than middle-aged adults. In the complete case analysis, the above-mentioned differences between middle-aged and older adults disappeared, but the direction of the effect size did not change. After the WL period, compared with men, women had less weight loss (adjusted difference for women vs men 1.78% [95% CI 1.12, 2.43], *p*<0.001) with greater fat-free mass and bone mass loss and smaller improvements in HbA_1c_, LDL-cholesterol and diastolic blood pressure. After the WM intervention, women had greater fat-free mass and bone mass loss and smaller improvements in HbA_1c_ and LDL-cholesterol, while they had greater improvements in fasting glucose, triacylglycerol (adjusted difference for women vs men −0.08 mmol/l [−0.11, −0.04], *p*<0.001) and HDL-cholesterol.

**Conclusions/interpretation:**

Older adults benefited less from a lifestyle intervention in relation to body composition and cardiometabolic health markers than younger adults, despite greater sustained weight loss. Women benefited less from a LED followed by a lifestyle intervention in relation to body weight and body composition than men. Future interventions targeting older adults or women should take prevention of fat-free mass and bone mass loss into consideration.

**Clinical trial registration number:**

ClinicalTrials.gov NCT01777893.

**Graphical abstract:**

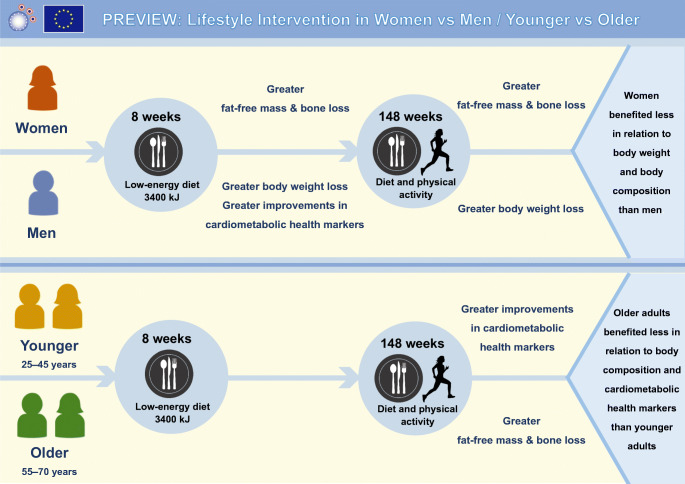

**Supplementary Information:**

The online version contains peer-reviewed but unedited supplementary material available at 10.1007/s00125-022-05716-3.

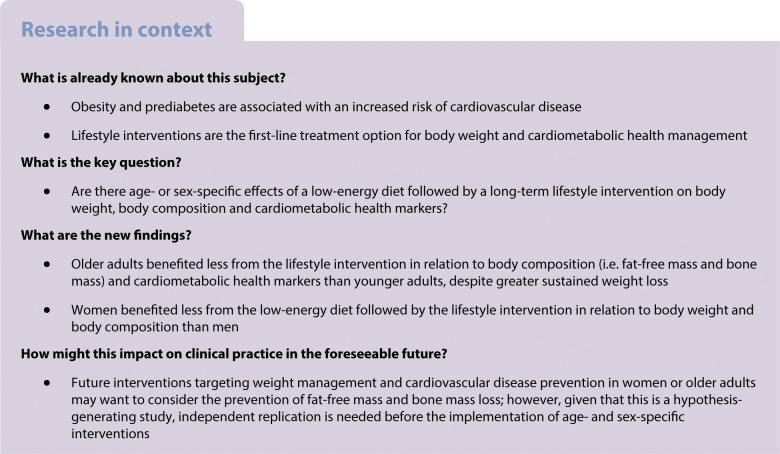



## Introduction

The global prevalence of obesity, which is associated with an increased risk of CVD [1, 2], is increasing. Clinical guidelines from the American Heart Association and ADA recommend weight loss of 5–8% for overweight and obese individuals to prevent CVD [3–6]. As the first-line treatment option for obesity, lifestyle interventions have been shown to aid weight loss and improve cardiometabolic health markers in several large-scale studies [7–10]. However, it is unclear whether the clinical guidelines benefit specific populations or whether personalised lifestyle interventions are needed.

Older adults (≥65 years) have attracted much attention, as they are highly likely to suffer from muscle and bone loss, sarcopenia and frailty during weight loss interventions, despite improvements in cardiometabolic health [11]. A systematic review suggested that lifestyle interventions were similarly effective at promoting weight loss and cardiometabolic health in older (≥60 years) and younger (<60 years) adults, but this conclusion was mainly based on short- or medium-term (≤2 years) studies and/or studies with a focus on older adults only [12]. A secondary analysis of the Action for Health in Diabetes (Look AHEAD) trial showed that, compared with middle-aged adults (45–64 years), older adults (65–76 years) lost more body weight and had comparable improvements in cardiometabolic health markers during a 4 year lifestyle intervention [13]. Few studies have compared changes in body composition and cardiometabolic health markers during a long-term (>2 years) lifestyle intervention in younger (25–45 years), middle-aged (46–54 years) and older (55–70 years) adults.

With regard to sex differences, a systematic review of RCTs of weight loss interventions reported that men demonstrated greater weight loss than women [14], whereas the concurrent change in cardiometabolic health markers is unknown. We have previously reported that women and men responded differently to an 8 week low-energy diet (LED) to induce rapid weight loss with regard to body weight and cardiometabolic health markers [15], but whether these effects will be long-lasting is unclear. A recent 2 year study found that there were no sex differences in intraorgan fat change and CVD risk after diet-induced weight loss [16], but the findings were limited by the small sample size.

The PREVention of diabetes through lifestyle interventions and population studies In Europe and around the World (PREVIEW) study was a 3 year, large-scale RCT of a lifestyle intervention for the prevention of diabetes in a large overweight population with prediabetes [17]. Type 2 diabetes and prediabetes have been demonstrated to be associated with an increased risk of CVD [18, 19] and the increased risk has been found to be mainly driven by abnormal levels of cardiometabolic health markers (e.g. blood pressure) [20]. Therefore, in the present observational study, we aimed to examine age- and sex-specific effects of a LED followed by a lifestyle-based weight maintenance (WM) intervention on body weight and cardiometabolic health markers in the PREVIEW participants. In addition, we compared the cumulative incidence of type 2 diabetes among age and sex groups.

## Methods

### Study design and participants

The PREVIEW study (ClinicalTrials.gov: NCT01777893) was a long-term, large-scale RCT conducted at eight intervention centres in Denmark, Finland, the Netherlands, the UK, Spain, Bulgaria, Australia and New Zealand. The detailed study design and main findings have been published [15, 17, 21]. Briefly, the primary outcome of the study was the impact of a high-protein/low-glycaemic index (HP/LGI) diet and a moderate-protein/moderate-glycaemic index (MP/MGI) diet on the risk of type 2 diabetes. The PREVIEW protocol was approved by the human ethics committee at each intervention centre (see electronic supplementary material [ESM] Table 1). The PREVIEW study was conducted in accordance with the Declaration of Helsinki.

Overweight or obese (BMI ≥25 kg/m^2^) adult participants aged 25–70 years with prediabetes were enrolled from June 2013 to April 2015. At the screening visit, an OGTT with 75 g glucose was conducted and those with impaired fasting plasma glucose (FPG) and/or impaired glucose tolerance were considered to have prediabetes, according to ADA criteria [22]. FPG and 2 h plasma glucose were measured using a glucose analyser. All eligible participants provided written informed consent.

### Interventions

The PREVIEW study consisted of two phases. Phase 1 was an 8 week rapid weight loss (WL) phase with a LED (3400 kJ/day) [15] and phase 2 was a 148 week WM phase. Those who failed to achieve the target weight reduction (>8% of initial body weight) were excluded. During the WM phase, participants were randomised into one of the four diet and physical activity (PA) combined intervention groups. Randomisation was stratified by age group (younger: 25–45 years; middle-aged: 46–54 years; older: 55–70 years) and sex (women and men). In defining the age range of each age group both the age classification from the World Health Organization [23] and the age range of menopause in women (44–54 years; menopause is associated with an increased risk of CVD [24]) were taken into consideration.

During the WM phase, participants were advised to consume an HP/LGI diet (25 E% [% energy] protein, 45 E% carbohydrates, GI <50) or an MP/MGI diet (15 E% protein, 55 E% carbohydrates, GI 56–70) combined with either high- or moderate-intensity PA. The high-intensity PA programme consisted of high-intensity PA (e.g. aerobics with very vigorous effort) for 75 min/week and the moderate-intensity PA programme consisted of moderate-intensity PA (e.g. conditioning exercises) for 150 min/week. Diets were consumed ad libitum without energy restriction. Counselling visits were conducted to improve diet and PA compliance, with decreasing frequency as the trial progressed [25]. Outcomes were collected at seven clinical investigation days (CIDs 1–7 at 0, 8, 26, 52, 78, 104 and 156 weeks, respectively); a detailed overview of the data collected at the different time points is provided in ESM Table 2. We allowed the following visit windows for data collection: at 8 weeks: −3 to +5 days; at 26 weeks: ±1 week; at 52 weeks: ±2 weeks; remaining time points: ±4 weeks. Adherence to the diets was evaluated using 4 day food records and adherence to the PA programmes was evaluated using 7 day accelerometry data.

This observational study is a post hoc, secondary analysis focusing on the secondary outcomes, including body weight and composition and cardiometabolic health markers. We merged all participants into one intervention group and reclassified them by sex and age range used in the original randomisation, because (1) there was no significant interaction of intervention arm and age or sex and (2) diet and PA compliance were lower than expected [17].

### Outcome measures

The measurements of body weight, waist circumference, fat mass, fat-free mass (FFM), bone mineral content (BMC), bone mineral density (BMD), FPG, 2 h plasma glucose, HbA_1c_, fasting triacylglycerol, HDL-cholesterol, LDL-cholesterol, systolic blood pressure (SBP) and diastolic blood pressure (DBP) were carried out as described previously [15, 17]. Briefly, all blood samples were drawn from the antecubital vein of participants in a fasting state (except for 2 h plasma glucose), stored at −80°C and transported to the Finnish Institute for Health and Welfare for analysis.

### Type 2 diabetes ascertainment

Type 2 diabetes was defined according to World Health Organization and ADA criteria [22, 26] and was diagnosed either (1) by an OGTT (FPG ≥7.0 mmol/l and/or 2 h plasma glucose ≥11.1 mmol/l) conducted at the intervention centres or (2) by a medical doctor. Most participants dropped out or completed the study by week 156, but some had a longer (>156 weeks) survival time because of the visit windows. We assumed that their last status was observed at 156 weeks.

### Statistical analysis

Linear mixed models were used to examine the associations of age and sex with changes in outcomes of interest during the 3 year lifestyle intervention from baseline to 156 weeks in all participants (available case analysis). Covariates that might influence the outcomes of interest [27–30] were added into the models (ESM Methods). Model 1 included age, sex, ethnicity, baseline BMI, baseline smoking status, baseline alcohol consumption, baseline energy intake and baseline PA, changes in energy intake and PA from baseline, baseline values of outcomes, intervention arm, time (categorical) and a two-way interaction of time and age group or sex as fixed covariates and participant identifier and intervention centre as random effects. Model 2 additionally included percentage weight loss from baseline as a fixed covariate when cardiometabolic health markers were added as a dependent variable. If the interaction term was significant, post hoc multiple comparisons with Bonferroni correction or pairwise comparisons (independent samples *t* tests) were conducted at each time point. Sensitivity analyses were conducted in those who completed the study or by additionally adjusting for carbohydrate, protein, fat and fibre intakes. For body composition outcomes, the models were additionally adjusted for light PA, moderate-to-vigorous PA, sedentary time and wear time.

The associations of percentage weight loss or weight regain with changes in cardiometabolic health markers during the rapid WL phase or the WM phase by age and sex were examined using linear mixed models including age, sex, ethnicity, baseline BMI, baseline smoking status, baseline alcohol consumption, baseline energy intake and PA, baseline values of outcomes, percentage weight loss from baseline and a two-way interaction of percentage weight loss and age group or sex as fixed covariates and intervention centre as a random effect. For weight regain, the models additionally included percentage regain weight and intervention arm as fixed covariates.

Cumulative incidence of type 2 diabetes by age and sex was calculated using the Kaplan–Meier method, without adjustment. Diabetes incidence was compared among age and sex groups using a time-dependent Cox hazards regression model adjusted for log_*e*_(time) × sex or age, ethnicity, baseline smoking status, baseline alcohol consumption, baseline BMI, baseline FPG, baseline 2 h plasma glucose, baseline PA and baseline energy intake, changes in PA and energy intake from baseline, intervention arm and intervention site as covariates. The proportional hazards assumption was evaluated using a Wald test of the interaction of time and age or sex.

Missing data, including dietary intake, PA and changes in outcomes of interest, were imputed using the expectation maximisation algorithm. The normality of residuals was determined using histograms and P–P plots. Data were analysed using IBM SPSS v28.0 software (Chicago, IL, USA) and OriginPro 2020 software (OriginLab, Northampton, MA, USA). The statistical test was two-sided and *p*<0.05 was considered statistically significant.

## Results

### Participants

**Fig. 1:**
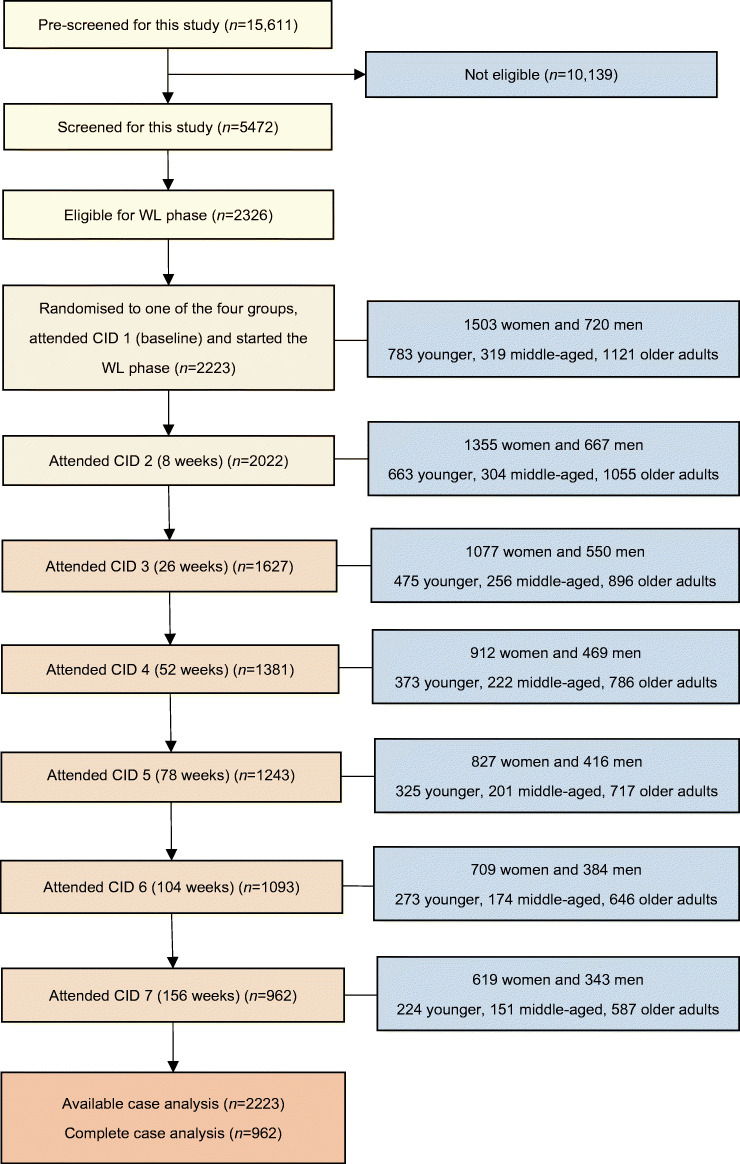
Study flow diagram. A total of 2224 participants started the weight loss phase, but one withdrew consent and requested data deletion. Younger adults: 25–45 years; middle-aged adults: 46–54 years; older adults: 55–70 years. To enable the data collection to be as complete as possible, we allowed the following visit windows for data collection: at 8 weeks: −3 to 5 days; at 26 weeks: ±1 week; at 52 weeks: ±2 weeks; remaining time points: ±4 weeks

A total of 2223 participants were included in the study (Fig. 1). Of these, 783 (35.2%) were younger, 319 (14.3%) were middle-aged and 1121 (50.4%) were older adults; 1503 (67.6%) were women and 720 (32.4%) were men. Participants’ baseline characteristics are shown in Table 1 and ESM Table 3. Dietary intake and PA by age and sex are shown in ESM Table 4.

**Table 1:** Baseline characteristics by age and sex

Variable	All participants (*n* = 2223)	Age group			Sex	
		Younger (*n* = 783)	Middle-aged (*n* = 319)	Older (*n* = 1121)	Women (*n* = 1503)	Men (*n* = 720)
Sociodemographics						
Women	1503 (67.6)	564 (72.0)	220 (69.0)	719 (64.1)	–	–
Age range, years	25–70	25–45	46–54	55–70	25–70	25–70
Age, years	55 (43, 61)	39 (34, 43)	50 (48, 52)	61 (57, 65)	53 (42, 60)	56 (44, 63)
Ethnicity						
White	1947 (87.6)	600 (76.6)	274 (85.9)	1073 (95.7)	1299 (86.4)	648 (90.0)
Other^a^	276 (12.4)	183 (23.4)	45 (14.1)	48 (4.3)	204 (13.6)	72 (10.0)
Smoking status						
No	1875 (84.3)	605 (77.3)	281 (88.1)	989 (88.2)	1264 (84.1)	611 (84.9)
Yes, but less than weekly	72 (3.2)	39 (5.0)	7 (2.2)	26 (2.3)	50 (3.3)	22 (3.1)
Yes, at least daily	239 (10.8)	124 (15.8)	27 (8.5)	88 (7.9)	169 (11.2)	70 (9.7)
Missing	37 (1.7)	15 (1.9)	4 (1.3)	18 (1.6)	20 (1.3)	17 (2.4)
Alcohol consumption						
No	718 (32.3)	319 (40.7)	106 (33.2)	293 (26.1)	571 (38.0)	147 (20.4)
Yes	1470 (66.1)	448 (57.2)	208 (65.2)	814 (72.6)	911 (60.6)	559 (77.6)
Missing	35 (1.6)	16 (2.0)	5 (1.6)	14 (1.2)	21 (1.4)	14 (1.9)
Anthropometry and body composition						
Body weight, kg	96.7 (84.7, 111.1)	103.0 (90.1, 118.8)	98.1 (84.7, 110.3)	92.7 (82.4, 105.6)	92.4 (81.6, 106.1)	104.8 (94.7, 118.9)
Height, m	1.67 (1.61, 1.75)	1.68 (1.63, 1.75)	1.66 (1.61, 1.74)	1.67 (1.61, 1.74)	1.64 (1.59, 1.68)	1.77 (1.73, 1.82)
Waist circumference, cm	110.4 (14.7)	111.2 (16.2)	109.8 (14.5)	110.1 (13.7)	107.5 (14.0)	116.7 (14.3)
BMI, kg/m^2^	33.9 (30.7, 38.5)	35.9 (31.7, 41.1)	34.0 (30.9, 38.8)	32.9 (30.0, 36.8)	34.3 (30.9, 39.3)	33.5 (30.5, 37.3)
Fat mass, kg	40.9 (33.4, 50.4)	44.5 (36.2, 54.9)	40.8 (33.8, 49.0)	38.6 (32.1, 47.3)	42.4 (34.7, 51.6)	37.4 (30.9, 46.3)
FFM, kg	54.0 (47.7, 64.1)	56.8 (49.8, 66.6)	53.6 (47.5, 64.1)	52.0 (46.2, 62.3)	49.7 (45.7, 54.9)	67.4 (61.7, 74.0)
BMC, g^b^	2720 (2401, 3136)	2886 (2544, 3271)	2756 (2414, 3170)	2600 (2300, 3000)	2550 (2318, 2800)	3336 (3000, 3662)
BMD, g/cm^2c^	1.3 (1.2, 1.3)	1.3 (1.2, 1.4)	1.3 (1.2, 1.3)	1.2 (1.1, 1.3)	1.2 (1.1, 1.3)	1.3 (1.2, 1.4)
Glucose metabolism						
FPG, mmol/l	6.2 (0.7)	5.9 (0.7)	6.2 (0.7)	6.3 (0.7)	6.1 (0.7)	6.3 (0.7)
2 h plasma glucose, mmol/l	7.7 (2.2)	7.2 (2.0)	7.9 (2.3)	7.9 (2.3)	7.6 (2.2)	7.7 (2.3)
HbA_1c_, mmol/mol	36.7 (4.0)	35.6 (4.1)	37.3 (4.1)	37.3 (3.7)	36.7 (3.9)	36.8 (4.1)
HbA_1c_, %	5.5 (0.4)	5.4 (0.4)	5.6 (0.4)	5.6 (0.3)	5.5 (0.4)	5.5 (0.4)
Lipid metabolism						
Triacylglycerol, mmol/l	1.3 (1.0, 1.8)	1.3 (1.0, 1.8)	1.3 (0.9, 1.7)	1.4 (1.1, 1.9)	1.3 (1.0, 1.7)	1.4 (1.1, 2.0)
Total cholesterol, mmol/l	5.2 (1.0)	5.0 (0.9)	5.2 (0.9)	5.3 (1.0)	5.3 (1.0)	5.0 (1.0)
HDL-cholesterol, mmol/l	1.2 (1.1, 1.4)	1.2 (1.0, 1.3)	1.3 (1.1, 1.4)	1.3 (1.1, 1.5)	1.3 (1.1, 1.5)	1.1 (1.0, 1.3)
LDL-cholesterol, mmol/l	3.2 (2.6, 3.8)	3.1 (2.5, 3.6)	3.3 (2.7, 3.8)	3.3 (2.7, 3.9)	3.3 (2.7, 3.8)	3.1 (2.6, 3.7)
Blood pressure						
SBP, mmHg	129.1 (15.9)	124.1 (14.7)	126.1 (14.3)	133.3 (15.9)	127.1 (16.0)	133.2 (14.7)
DBP, mmHg	78.7 (71.0, 85.3)	77.0 (69.0, 83.7)	78.2 (69.9, 84.5)	80.0 (72.7, 86.3)	77.3 (69.3, 84.3)	80.7 (74.3, 87.1)

Data are mean (SD), median (25th, 75th percentiles) or *n* (%)

^a^Includes Asian, black, Arabic, Hispanic and other participants. χ^2^ test was performed based on full categories including white, Asian, black, Arabic, Hispanic and other participants

^b^Data available for 614 of 783 younger participants, 227 of 319 middle-aged participants and 639 of 1121 older participants or 1037 of 1503 women and 443 of 720 men from Denmark, Spain, Bulgaria, Australia and New Zealand

^c^Data available for 419 of 783 younger participants, 221 of 319 middle-aged participants and 476 of 1121 older participants or 759 of 1503 women and 357 of 720 men from Denmark, Spain, Australia and New Zealand

### Changes in body weight and cardiometabolic health markers by age

**Fig. 2:**
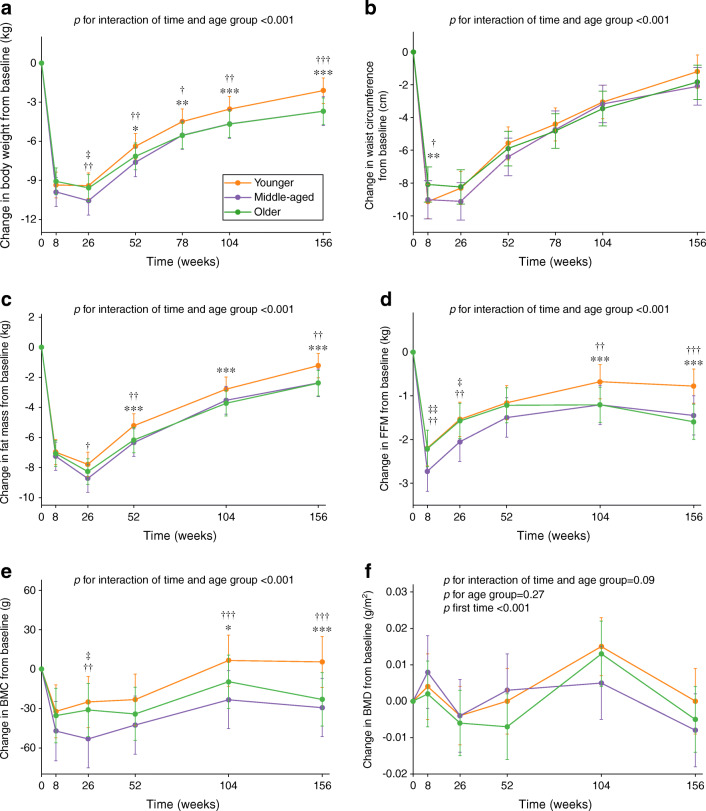
Changes in anthropometry and body composition from baseline by age group (*n*=2223). Values are estimated marginal mean (95% CI) changes from baseline in body weight (**a**), waist circumference (**b**), fat mass (**c**), FFM (**d**), BMC (**e**) and BMD (**f**). Younger adults: 25–45 years; middle-aged adults: 46–54 years; older adults: 55–70 years. Analyses were performed using a linear mixed model including sex, age, ethnicity, baseline BMI, baseline smoking status, baseline alcohol consumption, baseline values of the outcome being considered, baseline energy intake and PA, time-varying changes in energy intake and PA from baseline, intervention arm, time and interaction of time and age group or sex as fixed covariates and participant identifier and intervention centre as random effects. Post hoc multiple comparisons with Bonferroni adjustment were performed to compare age groups at each time point, where appropriate. Older vs younger adults *p<0.05, **p<0.01 and ***p<0.001; middle-aged vs younger adults ^†^*p*<0.05, ^††^*p*<0.01 and ^†††^*p*<0.001; older vs middle-aged adults ^‡^*p*<0.05 and ^‡‡^*p*<0.01. BMC data were based on 614 younger, 227 middle-aged and 639 older participants from Denmark, Spain, Bulgaria, Australia and New Zealand. BMD data were based on 419 younger, 221 middle-aged and 476 older participants from Denmark, Spain, Australia and New Zealand

In the available case analysis, there were no differences in weight loss among age groups at 8 weeks, whereas middle-aged and older adults had greater sustained weight loss than younger adults during the WM phase at 78, 104 and 156 weeks (adjusted mean difference for older vs younger adults at 156 weeks −1.58 kg [95% CI −2.27, −0.89], *p*<0.001; −1.25% [95% CI −1.92, −0.58], *p*<0.001; Fig. 2 and ESM Fig. 1, respectively). Older and younger adults lost similar FFM (kg and %) and BMC at 8 weeks, but older adults had less FFM and BMC regain than younger adults during the WM phase at 104 and 156 weeks. In the complete case analysis, middle-aged adults had greater weight loss (kg) at 156 weeks than younger adults, whereas there were no differences in weight loss (%) between the two age groups at 156 weeks (ESM Fig. 2). The significant differences in FFM and BMC remained after additionally adjusting for PA type.

**Fig. 3:**
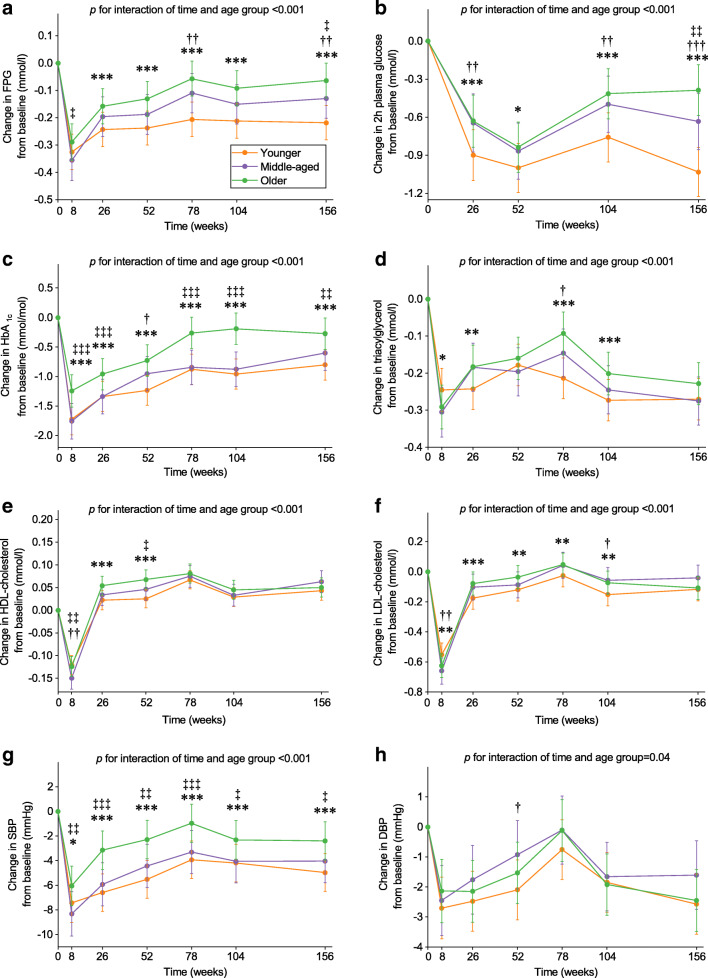
Weight loss-adjusted changes in cardiometabolic health markers from baseline by age group (*n*=2223). Values are estimated marginal mean (95% CI) changes from baseline in FPG (**a**), 2 h plasma glucose (**b**), HbA_1c_ (**c**), triacylglycerol (**d**), HDL-cholesterol (**e**), LDL-cholesterol (**f**), SBP (**g**) and DBP (**h**). Younger adults: 25–45 years; middle-aged adults: 46–54 years; older adults: 55–70 years. Analyses were performed using a linear mixed model including sex, age, ethnicity, baseline BMI, baseline smoking status, baseline alcohol consumption, baseline values of the outcome being considered, time-varying percentage weight loss from baseline, baseline energy intake and PA, time-varying changes in energy intake and PA from baseline, intervention arm, time and interaction of time and age group as covariates and participant identifier and intervention centre as random effects. Post hoc multiple comparisons with Bonferroni adjustment were performed to compare age groups at each time point. Older vs younger adults *p<0.05, **p<0.01 and ***p<0.001; middle-aged vs younger adults ^†^*p*<0.05, ^††^*p*<0.01 and ^†††^*p*<0.001; older vs middle-aged adults ^‡^*p*<0.05, ^‡‡^*p*<0.01 and ^‡‡‡^*p*<0.001

In the available case analysis, compared with younger adults, older adults had smaller decreases in HbA_1c_ and SBP at 8 weeks and they maintained greater improvements in these outcomes and in 2 h plasma glucose during the whole WM phase (ESM Fig. 3). Older adults had greater decreases in triacylglycerol than younger adults at 8 weeks, but the differences disappeared at 156 weeks. After adjustment for weight loss, the above-mentioned significant differences remained (adjusted mean difference in 2 h plasma glucose for older vs younger adults at 156 weeks 0.65 mmol/l [95% CI 0.50, 0.80], *p*<0.001; adjusted mean difference in SBP for older vs younger adults at 156 weeks 2.57 mmHg [95% CI 1.37, 3.77], *p*<0.001; Fig. 3). In addition, smaller decreases in FPG and 2 h plasma glucose in middle-aged vs younger adults and smaller decreases in FPG, 2 h plasma glucose, HbA_1c_ and SBP in older vs middle-aged adults were observed at 156 weeks. The above-mentioned results remained robust after adjusting for dietary intake. In the complete case analysis, there were no significant differences in the above-mentioned outcomes between middle-aged and older adults at 156 weeks, but the direction of effect was the same (ESM Fig. 4).

### Changes in body weight and cardiometabolic health markers by sex

**Fig. 4:**
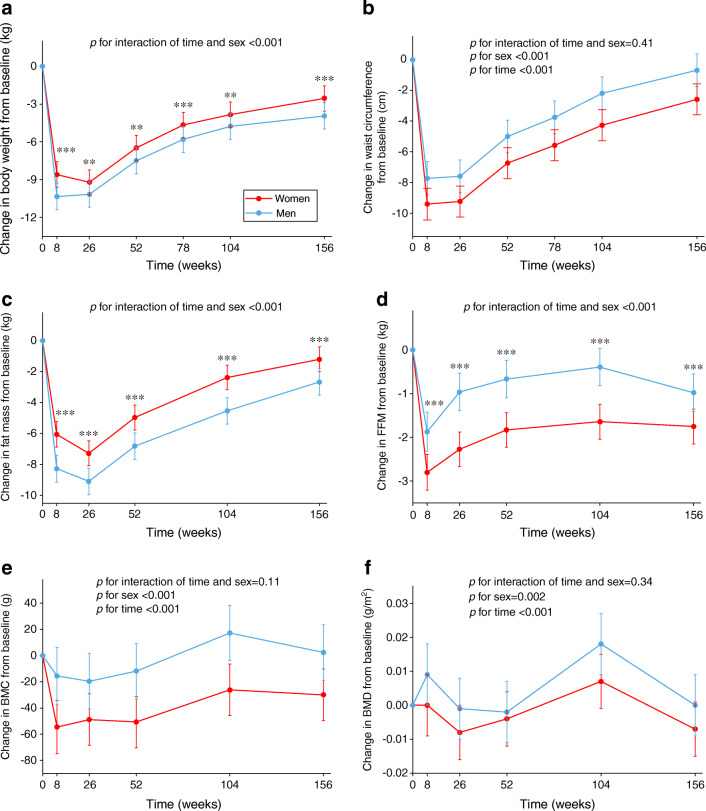
Changes in anthropometry and body composition from baseline in women and men (*n*=2223). Values are estimated marginal mean (95% CI) changes from baseline in body weight (**a**), waist circumference (**b**), fat mass (**c**), FFM (**d**), BMC (**e**) and BMD (**f**). Analyses were performed using a linear mixed model including sex, age, ethnicity, baseline BMI, baseline smoking status, baseline alcohol consumption, baseline values of the outcome being considered, baseline energy intake and PA, time-varying changes in energy intake and PA from baseline, intervention arm, time and interaction of time and age group or sex as fixed covariates and participant identifier and intervention centre as random effects. Post hoc pairwise comparisons (independent samples *t* tests) were performed to compare women and men at each time point, where appropriate. Women vs men **p<0.01 and ***p<0.001. BMC data were based on 1037 women and 443 men from Denmark, Spain, Bulgaria, Australia and New Zealand. BMD data were based on 759 women and 357 men from Denmark, Spain, Australia and New Zealand

In the available case analysis, compared with men, women lost less body weight (adjusted mean difference at 8 weeks 1.74 kg [95% CI 1.07, 2.41], *p*<0.001; 1.12% [95% CI 0.47, 1.78], *p*<0.001; Fig. 4 and ESM Fig. 5, respectively) but more FFM (kg and %) at 8 weeks and had less sustained weight loss (adjusted mean difference at 156 weeks 1.39 kg [95% CI 0.71, 2.06], *p*<0.001; 1.78% [95% CI 1.12, 2.43], *p*<0.001) and less change in FFM during the WM phase. In addition, women lost more BMC and BMD than men over 3 years. The above-mentioned results remained robust in the complete case analysis. The significant differences in FFM and BMC remained after additionally adjusting for PA type.

**Fig. 5:**
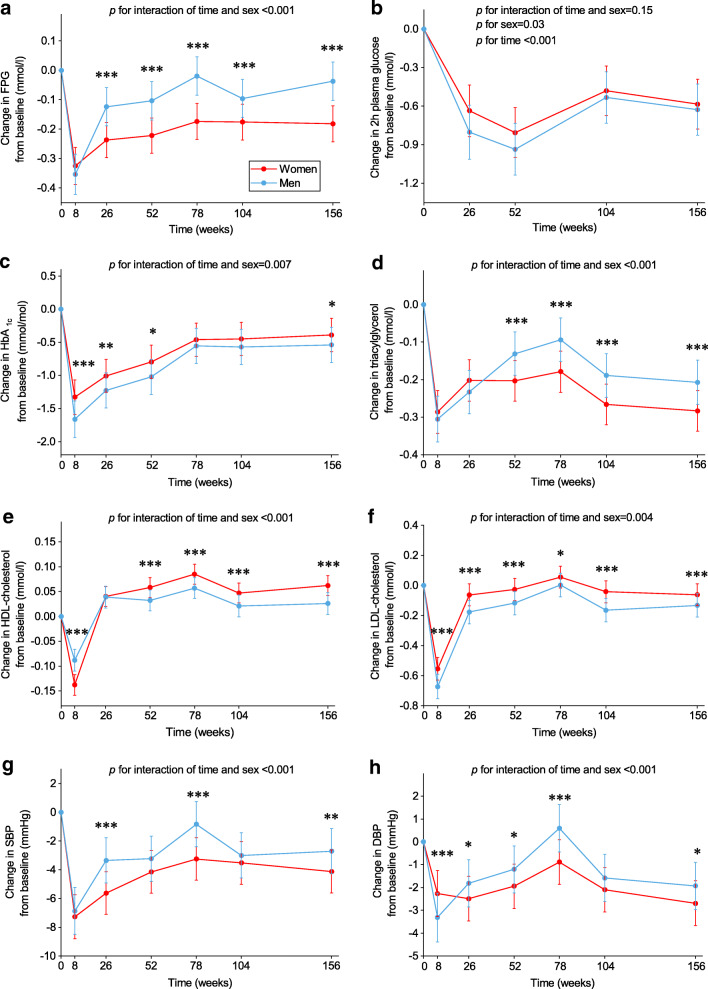
Weight-adjusted changes in cardiometabolic health markers from baseline in women and men (*n*=2223)**.** Values are estimated marginal mean (95% CI) changes from baseline in FPG (**a**), 2 h plasma glucose (**b**), HbA_1c_ (**c**), triacylglycerol (**d**), HDL-cholesterol (**e**), LDL-cholesterol (**f**), SBP (**g**) and DBP (**h**). Analyses were performed using a linear mixed model including sex, age, ethnicity, baseline BMI, baseline smoking status, baseline alcohol consumption, baseline values of the outcome being considered, time-varying percentage weight loss from baseline, baseline energy intake and PA, time-varying changes in energy intake and PA from baseline, intervention arm, time and interaction of time and sex as fixed covariates and participant identifier and intervention centre as random effects. Post hoc pairwise comparisons (independent samples *t* tests) were performed to compare women and men at each time point, where appropriate. Women vs men *p<0.05, **p<0.01 and ***p<0.001

In the available case analysis, compared with men, women had smaller decreases in FPG, HbA_1c_, triacylglycerol, LDL-cholesterol and DBP and greater decreases in HDL-cholesterol at 8 weeks (ESM Fig. 6). After the WM phase, women had greater improvements in FPG and HDL-cholesterol than men, while they had smaller improvements in HbA_1c_ and LDL-cholesterol. After adjustment for percentage weight loss, the significant differences in HbA_1c_, LDL-cholesterol, HDL-cholesterol and DBP between women and men remained at 8 weeks (Fig. 5). In this analysis, after the WM phase, women had greater improvements in FPG, triacylglycerol, HDL-cholesterol, SBP and DBP than men (adjusted mean difference at 156 weeks in FPG −0.15 mmol/l [95% CI −0.18, −0.11], *p*<0.001; SBP −1.41 mmHg [95% CI −2.34, −0.48], *p*=0.003; triacylglycerol −0.08 mmol/l [95% CI −0.11, −0.04], *p*<0.001; Fig. 5), while they had smaller improvements in HbA_1c_ and LDL-cholesterol. The above-mentioned results remained robust after adjusting for dietary intake. In the complete case analysis, there were no significant differences in SBP and DBP between women and men at 156 weeks, but the direction of effect was the same (ESM Fig. 7).

### Associations of weight change with cardiometabolic health markers

Rapid weight loss was associated with greater improvements in SBP in older adults than younger adults (ESM Table 5). Weight loss was associated with smaller improvements in FPG, triacylglycerol, HDL-cholesterol, LDL-cholesterol, SBP and DBP in women than men. During the WM phase, weight regain was associated with more adverse FPG, HbA_1c_ and SBP in older vs younger adults.

### Type 2 diabetes incidence

**Fig. 6:**
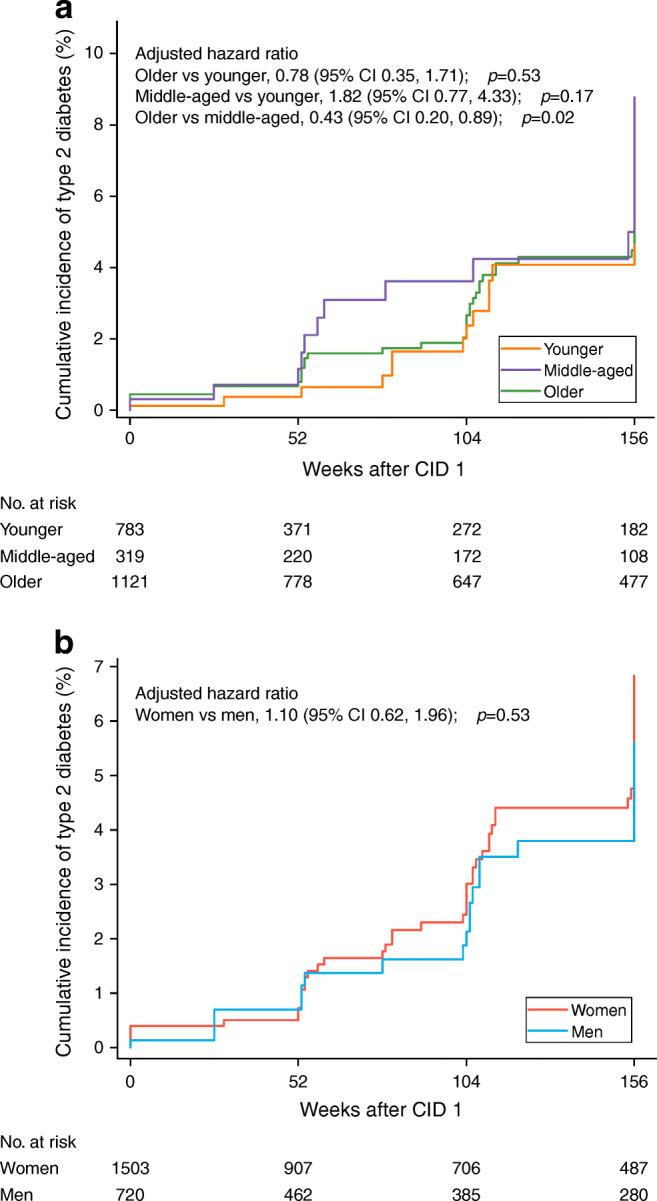
Cumulative incidence of type 2 diabetes by age and sex (*n*=2223). Values are cumulative incidence of diabetes by age (**a**) and sex (**b**) at each time point. Diabetes was diagnosed by an OGTT with 75 g glucose or by a medical doctor. Cumulative incidence was calculated using the Kaplan–Meier method, without adjustment. The incidence of diabetes was compared among age groups or between women and men using a time-dependent Cox hazards regression model adjusted for log_*e*_(time) × age or sex, ethnicity, baseline smoking status, baseline alcohol consumption, baseline BMI, baseline FPG, baseline 2 h plasma glucose, baseline PA and baseline energy intake, changes in PA and energy intake from baseline, intervention arm and intervention site as covariates

The total number of cases of type 2 diabetes was 69 (seven during the rapid WL phase and 62 during the WM phase; 13 younger, 14 middle-aged and 42 older adults; 48 women and 21 men). The 3 year cumulative incidence was 4.6% in younger, 8.8% in middle-aged and 6.6% in older adults; and 6.8% in women and 5.6% in men (Fig. 6). The adjusted hazard ratio was 0.43 (95% CI 0.20, 0.89) for older vs middle-aged adults (*p*=0.02).

## Discussion

We found that the cardiometabolic benefits of a LED followed by a lifestyle intervention differed by age and sex in overweight adults with prediabetes. Older adults benefited less from a lifestyle-based WM intervention in relation to body composition and cardiometabolic health markers than younger adults, despite greater sustained weight loss. Women benefited less from the lifestyle intervention in relation to body weight and composition and cardiometabolic health markers than men. After the lifestyle-based WM phase, women showed greater improvements in fasting glucose, triacylglycerol and HDL-cholesterol, and smaller improvements in HbA_1c_ and LDL-cholesterol. Older adults had a lower incidence of type 2 diabetes at the end of the study than middle-aged adults.

Several potential mechanisms may explain the observed age and sex differences in the present study. Ageing may lead to the redistribution of adipose tissue, from subcutaneous to visceral depots [31]. Distribution of adipose tissue is also affected by sex [32]. Epidemiological studies have confirmed the association of visceral adiposity with deteriorating metabolic outcomes, whereas subcutaneous adipose tissue has been found to be associated with protective properties [33]. In addition, epigenetic age acceleration was found to be positively correlated with glucose and the triacylglycerol–glucose index [34].

In the present study, older adults had greater sustained weight loss than younger adults, which is in agreement with the findings from the secondary analyses of the Weight Loss Maintenance trial and the Look AHEAD trial [13, 35]. However, among those participating in the adapted Diabetes Prevention Program (DPP) lifestyle intervention, there were no differences in weight loss between older and younger adults [36]. The conflicting results may be attributed to differences in study duration and the age range of groups compared. In the adapted DPP, the intervention lasted only 10 months, whereas in our study we did not find significant differences in weight loss between younger and older adults until 52 weeks. In addition, in the adapted DPP, participants were divided into only two age groups (<65 vs ≥65 years), whereas in our study we compared three age groups.

Notably, the above-mentioned studies did not report body composition data and the present study therefore adds to the evidence base by exploring long-term changes in body composition among age groups. Less weight regain but also less regain of FFM and BMC after rapid weight loss were observed in older adults than in younger adults, which suggests that future weight management programmes designed for older adults should take both weight loss and prevention of FFM and BMC loss into consideration. Regarding prevention of FFM and BMC loss, an RCT demonstrated that resistance exercise was associated with a lower weight loss-induced decrease in BMD in older adults [37]. Moreover, systematic reviews have suggested that resistance exercise is effective in the prevention of osteoporosis in older adults [38] and that exercise is also effective in the prevention of FFM loss in middle-aged and older adults after moderate energy restriction-induced weight loss [39]. In the PREVIEW study, participants were not advised to focus on resistance exercise during the WM phase, because the aim of the study was to compare the effect of intensity levels rather than types of exercise. For preservation of BMC and FFM, future weight management programmes might consider including other types of PA (e.g. resistance exercise).

Many previous studies have explored the effects of lifestyle interventions on cardiometabolic health markers in older adults [12, 36] and the associations of weight loss with cardiometabolic health markers [9, 40–42], whereas few have compared changes in cardiometabolic health markers across age groups. In the present study, older adults had smaller improvements in cardiometabolic health markers than younger adults during the WM phase, especially at the end of the study, although they had a worse metabolic profile at baseline (a larger potential for improvement) and greater sustained weight loss. The differences at the end of the study between younger and older adults still remained after adjustment for weight loss, which suggests that age per se may also influence cardiometabolic health markers. In addition, we found that older adults, who had more adverse metabolic profiles at baseline, had a lower incidence of type 2 diabetes at the end of the study than middle-aged adults. This might be partly explained by the effect of menopause on the risk of type 2 diabetes in middle-aged women [43]. Selection bias caused by a high attrition rate may be another explanation.

In terms of sex differences in changes in body weight and body composition, in a previous PREVIEW publication we reported that women lost less body weight but more FFM and BMC than men during the rapid weight loss phase [15]; in the present study these patterns were the same during the WM phase. Our findings on sex differences in weight loss are in line with a meta-analysis of six lifestyle-based weight loss RCTs [14] and a recent study on sex differences in intraorgan fat levels and hepatic lipid metabolism [16]. In addition, a systematic review suggested that men tend to lose more weight with intensive low-fat reducing diets and PA programmes than women [44]. However, a review argued that the observed sex differences in weight loss are attributable to greater initial body weight or a greater degree of energy restriction in men, instead of inherent sex differences [32]. In the present study, the difference in weight loss still remained after adjustment for baseline body weight and change in energy intake from baseline. Considering body composition, in agreement with our findings, Evans et al [45] also found that men lost body fat (%) more effectively than women in a 1 year weight loss trial including dietary interventions and exercise guidance. In addition, Tirosh et al [46] found that women had a greater increase in the fat mass/FFM ratio and a greater reduction in BMD than men during a lifestyle-based weight loss intervention. Accordingly, it may be important for women to prevent FFM and BMC loss when participating in weight management programmes. Current data show that dietary protein may affect body composition [47], but in the present study the sex-specific differences in fat mass and BMC remained significant even after adjusting for protein intake. Moreover, our findings were also independent of PA type (i.e. light PA and moderate-to-vigorous PA). Previous studies have found that other types of PA (e.g. resistance exercise and aerobic exercise) may have different effects on body composition [47] and future studies should therefore investigate whether sex influences these effects.

A recent review suggested that sex may be an important factor in determining the effect of dietary or lifestyle interventions on cardiometabolic health [48]. In particular, Perreault et al [49] found that in the DPP lifestyle cohort, among those who lost >3% of their body weight, men appeared to have greater decreases in cardiometabolic health markers than women. However, this study did not include a diet-induced rapid WL phase and DPP investigators were therefore not able to observe sex differences in changes in cardiometabolic health markers during a rapid WL phase and a WM phase. In the present study, we showed that men benefited more in terms of body weight and composition and cardiometabolic health markers from the diet-induced WL phase. In addition, weight loss was associated with greater improvements in cardiometabolic health markers in men than women during the rapid WL phase. These findings suggest that the LED and rapid weight loss may be more effective in CVD prevention in men than in women.

The present study has some strengths. The large sample size and representative populations from eight countries could be considered a strength. Because of the wide age range included, we were able to compare differences in outcomes among younger, middle-aged and older adults. In addition, unlike short-term studies, our study was able to address longer term comorbidities associated with obesity and related diseases such as CVD. The present study also has limitations. First, the attrition rate was higher than expected, which resulted in a high percentage of missing data. To reduce the bias, we imputed the missing data and carried out a complete case analysis. Most of our findings in younger vs older adults and women vs men remained robust in the complete case analysis. Moreover, the significant differences in younger vs older adults also remained in weight-adjusted models. Significant differences between middle-aged adults and other age groups, however, disappeared in the complete case analysis, but the direction of effect was the same. This may be because the differences were small and were not detectable in the small sample size (completers). Finally, the design and statistical analysis of the present secondary analysis were not prespecified and the baseline characteristics of subgroups were not precisely balanced. We adjusted for baseline BMI and outcomes, but it was not possible to completely remove some other participant-specific baseline characteristics, especially CVD and type 2 diabetes risk. These unmeasured and unadjusted confounders may have influenced the results. Taken together, given the existence of bias, our findings should be interpreted with caution and warrant further replication in an independent study.

In conclusion, this observational study found that, in a large population of overweight adults with prediabetes, the cardiometabolic benefits of a LED followed by a lifestyle intervention differed by age and sex. Older adults benefited less from the lifestyle intervention in relation to body composition and cardiometabolic health markers than younger adults. Women benefited less from the LED followed by the lifestyle intervention in relation to body weight and body composition than men. Our findings suggest that future weight management programmes designed for older adults or women may want to consider the prevention of FFM and bone mass loss. However, given that this is a hypothesis-generating study, independent replication is needed before the implementation of age- and sex-specific interventions.

## Supplementary information

ESM 1 (PDF 485 kb)

## Data Availability

The datasets analysed during the current study are available from the corresponding author on reasonable request.

## Abbreviations

BMC: Bone mineral content
BMD: Bone mineral density
CID: Clinical investigation day
DBP: Diastolic blood pressure
DPP: Diabetes Prevention Program
FFM: Fat-free mass
FPG: Fasting plasma glucose
HP/LGI: High-protein/low-glycaemic index diet
LED: Low-energy diet
Look AHEAD: Action for Health in Diabetes
MP/MGI: Moderate-protein/moderate-glycaemic index diet
PA: Physical activity
PREVIEW: PREVention of diabetes through lifestyle interventions and population studies In Europe and around the World
SBP: Systolic blood pressure
WL: Weight loss (period)
WM: Weight maintenance (intervention)
