# Supplementary material for: Age- and sex-specific effects of a long-term lifestyle intervention on body weight and cardiometabolic health markers in adults with prediabetes: results from the diabetes prevention study PREVIEW
Source: Diabetologia. 2022 May 25;65(8):1262–77. doi: 10.1007/s00125-022-05716-3 (PMC9283166; doi:10.1007/s00125-022-05716-3)
Supplement: Supplementary file 1 — (PDF 485 kb) [file 125_2022_5716_MOESM1_ESM.pdf]

Electronic Supplementary Material

Age- and sex-specific effects of a long-term lifestyle intervention on body weight and cardiometabolic health markers in adults with prediabetes: results from the diabetes prevention study PREVIEW

ESM Methods

Statistical analysis

In the present study, the following factors, which may influence the outcomes of interest, were considered covariates. Ethnicity (White, Asian, Black, Arabic, Hispanic, or other), smoking status (daily, less than weekly, or no smoking) and alcohol drinking (yes or no) was self-reported by participants at baseline. Dietary intake was assessed using four day food records. Total physical activity and different types of physical activity (i.e., light physical activity, moderate to vigorous moderate physical activity and sedentary time) were assessed using a hip-worn ActiSleep+ (ActiGraph LLC, Pensacola, FL) accelerometer. Total physical activity was estimated using mean activity counts (counts/min) over valid wear time. Troiano cut points were used to assess time (min/day) spent at different types of physical activity (i.e. sedentary time <100 counts/min, light physical activity 100–2020 counts/min, moderate physical activity <2020 counts/min and vigorous physical activity <5999 counts/min. Moderate to vigorous physical activity was the sum of moderate and vigorous activity physical activity.

ESM Table 1. Human Ethics Committees for each intervention centre

Intervention centers	Human Ethics Committees
Denmark (University of Copenhagen)	The Research Ethics Committees of the Capital Region
Finland (University of Helsinki)	Coordinating Ethical Committee of HUS (Helsinki and Uusimaa Hospital District)
The Netherlands (University of Maastricht)	Medical Ethics Committee of the Maastricht University Medical Centre
The U.K. (University of Nottingham)	UK National Research Ethics Service (NRES) and East Midlands (Leicester) Ethics Committee
Spain (University of Navarra)	Research Ethics Committee of the University of Navarra
Bulgaria (Medical University of Sofia)	Commission on Ethics in Scientific Research with the Medical University-Sofia (KENIMUS)
Australia (University of Sydney)	The University of Sydney, Human Research Ethics Committee (HREC)
New Zealand (University of Auckland)	Health and Disability Ethics Committees (HDEC)

ESM Table 2. Overview of data collection over 3 years at different time points

Outcome	CID1 (0 weeks)	CID2 (8 weeks)	CID3 (26 weeks)	CID4 (52 weeks)	CID5 (78 weeks)	CID6 (104 weeks)	CID7 (156 weeks)
Socio-demographics (age, sex, ethnicity, baseline smoking status and baseline alcohol drinking)	×						
Anthropometry (body weight and waist circumference)	×	×	×	×	×	×	×
Body composition (fat mass, fat-free mass, bone mineral content and bone mineral density)	×	×	×	×		×	×
Glucose metabolism (fasting plasma glucose and HbA_{1c})	×	×	×	×		×	×
Glucose metabolism (2h plasma glucose)	×		×	×		×	×
Blood pressure (systolic blood pressure and diastolic blood pressure)	×	×	×	×		×	×
Lipid metabolism (total cholesterol, high-density lipoprotein cholesterol and fasting triglycerides)	×	×	×	×		×	×
Dietary intake[a]	×		×	×		×	×
Physical activity[a]	×		×	×		×	×

[a] Baseline dietary intake and physical activity and changes in dietary intake and physical activity from baseline were calculated and added to the linear mixed model. The macronutrient composition of the low-energy diet (3400 kJ/day⁻, protein 43.7 E%, carbohydrate 41.2 E%, fat 15.1 E%, fibre 13.3 g/day) will be used to estimate dietary intake at 8 weeks. Physical activity at 0 weeks was used to estimate physical activity at 8 weeks, assuming that physical activity did not change from during the weight loss phase. Average dietary intake at 52 and 104 weeks was used to estimate dietary intake at 78 weeks. Average physical activity at 52 and 104 weeks was used to estimate physical activity at 78 weeks. CID, clinical investigation day.

ESM Table 3. Ethnicity by sex and age

	All participants (n=2223)	Age group			Sex	
		**Younger (n=783)**	**Middle-aged (n=319)**	**Older (n=1121)**	**Women (n=1503)**	**Men (n=720)**
White	1947 (87.6%)	600 (76.6%)	274 (85.9%)	1073 (95.7%)	1299 (86.4%)	648 (90.0%)
Asian	60 (2.7%)	40 (5.1%)	8 (2.5%)	12 (1.1%)	39 (2.6%)	21 (2.9%)
Black	40 (1.8%)	22 (2.8%)	7 (2.2%)	11 (1.0%)	30 (2.0%)	10 (1.4%)
Arabic	5 (0.2%)	1 (0.1%)	3 (0.9%)	1 (0.1%)	3 (0.2%)	2 (0.3%)
Hispanic	44 (2.0%)	28 (3.6%)	12 (3.8%)	4 (0.4%)	34 (2.3%)	10 (1.4%)
Other	127 (5.7%)	92 (11.7%)	15 (4.7%)	20 (1.8%)	98 (6.5%)	29 (4.0%)

Data are n (%). Younger adults: 25−45 years; middle-aged adults: 46−54 years; older adults: 55−70 years.

ESM Table 4. Dietary intake and physical activity by age and sex (n=2223)

	Group		0 weeks	26 weeks	52 weeks	104 weeks	156 weeks	*p* for interaction of group and time	*p* for group main effect	*p* for time main effect
Carbohydrate, E%	Age	Younger	41 (7)a	40 (6)a	41 (6)a	40 (6)a	40 (5)a	<0.001	–	–
		Middle-aged	39 (8)b	40 (7)a	40 (6)a	40 (7)a	39 (7)a			
		Older	39 (6)b	40 (7)a	40 (6)a	40 (6)a	39 (6)a			
	Sex	Women	41 (7)a	40 (6)a	40 (6)a	40 (6)a	40 (6)a	0.004	–	–
		Men	39 (7)b	40 (7)a	40 (7)a	40 (6)a	39 (7)a			
Protein, E%	Age	Younger	17 (4)	20 (3)	20 (3)	20 (3)	20 (3)	0.07	0.002	<0.001
		Middle-aged	18 (4)	21 (4)	20 (3)	20 (4)	21 (4)			
		Older	18 (3)	21 (4)	20 (3)	20 (3)	20 (3)			
	Sex	Women	17 (3)	20 (4)	20 (3)	20 (3)	20 (3)	0.18	<0.001	<0.001
		Men	18 (4)	21 (4)	20 (3)	20 (3)	20 (4)			
Fat, E%	Age	Younger	38 (6)	35 (6)	35 (5)	36 (5)	36 (4)	0.85	<0.001	<0.001
		Middle-aged	37 (6)	34 (6)	35 (6)	35 (5)	35 (5)			
		Older	37 (6)	33 (6)	34 (6)	35 (6)	35 (5)			
	Sex	Women	37 (6)	34 (6)	35 (5)	35 (5)	36 (5)	0.25	<0.001	<0.001
		Men	36 (6)	33 (6)	34 (6)	34 (6)	35 (5)			
Fibre, g/day	Age	Younger	22 (8)	22 (6)	22 (6)	21 (5)	21 (5)	0.10	<0.001	<0.001
		Middle-aged	22 (8)	22 (6)	22 (7)	21 (6)	21 (6)			
		Older	22 (8)	23 (7)	23 (7)	22 (6)	21 (7)			
	Sex	Women	21 (7)	22 (6)	22 (6)	21 (5)	21 (6)	0.17	<0.001	<0.001
		Men	23 (9)	23 (7)	24 (7)	22 (6)	22 (6)			
Energy, kcal/day	Age	Younger	2221 (668)a	1673 (378)a	1694 (419)a	1651 (414)a	1654 (379)a	<0.001	–	–
		Middle-aged	2111 (575)b	1657 (403)a	1653 (392)a	1630 (389)a	1593 (348)a			
		Older	2025 (553)c	1649 (399)a	1636 (408)a	1599 (363)a	1594 (371)a			
	Sex	Women	2024 (569)b	1601 (365)b	1607 (383)b	1575 (349)b	1571 (357)b	<0.001	–	–
		Men	2278 (642)a	1780 (418)a	1766 (444)a	1719 (439)a	1707 (385)a			
Total physical activity, counts/min	Age	Younger	304 (99)a	335 (98)a	329 (92)a	318 (85)a	308 (87)a	0.05	–	–
		Middle-aged	298 (101)ab	342 (120)a	339 (116)a	325 (110)a	311 (108)a			
		Older	281 (108)b	319 (115)b	306 (114)b	300 (109)b	284 (113)b			
	Sex	Women	288 (93)a	320 (98)b	314 (94)b	305 (90)b	289 (87)b	0.003	–	–
		Men	299 (126)a	343 (132)a	330 (131)a	321 (122)a	313 (131)a			

Data are unadjusted mean (SD). Younger adults: 25−45 years; middle-aged adults: 46−54 years; older adults: 55−70 years. Analyses were performed based on imputed data and using a linear mixed model with repeated measures adjusted for sex or age and time as fixed effects and participant identifier and intervention centre as random effects. Time by age or sex interaction terms were added. Post hoc multiple comparisons with Bonferroni adjustment or pairwise comparisons (independent samples *t* test) were performed to compare age groups or women and men at each time point, where appropriate. Values with the different lowercase letters (a, b, and c) in a column are significantly different, $p<0.05$.

ESM Table 5. Associations of weight change with changes in cardiometabolic health markers (n=2223)

	Mean changes (95% CI) in all participants[a]	*p* value[b]	Relative mean changes (95% CI) by age[d]			Relative mean changes (95% CI) by sex[d]	
			Younger[c]	Middle-aged	Older	Men[c]	Women
Percentage weight loss (each 10%) during rapid weight loss							
Fasting plasma glucose, mmol/l	-0.38 (-0.445, -0.32)	<0.001	0 (Ref.)	-0.005 (-0.19, 0.18)	-0.10 (-0.21, 0.01)	0 (Ref.)	0.17 (0.06, 0.28)e
HbA_{1c}, mmol/mol	-1.16 (-1.38, -0.94)	<0.001	0 (Ref.)	0.11 (-0.58, 0.80)	-0.19 (-0.61, 0.23)	0 (Ref.)	0.14 (-0.26, 0.55)
Triacylglycerol, mmol/l	-0.27 (-0.32, -0.21)	<0.001	0 (Ref.)	-0.08 (-0.25, 0.09)	-0.006 (-0.11, 0.10)	0 (Ref.)	0.17 (0.07, 0.26)e
HDL-cholesterol, mmol/l	-0.07 (-0.09, -0.05)	<0.001	0 (Ref.)	0.08 (0.02, 0.14)e	0.002 (-0.03, 0.04)	0 (Ref.)	-0.06 (-0.09, -0.02)e
LDL-cholesterol, mmol/l	-0.42 (-0.48, -0.35)	<0.001	0 (Ref.)	0.16 (-0.04, 0.37)	0.03 (-0.10, 0.15)	0 (Ref.)	0.12 (0.001, 0.24)e
Systolic blood pressure, mmHg	-4.49 (-5.96, -3.02)	<0.001	0 (Ref.)	-0.15 (-4.32, 4.72)	-3.10 (-5.87, -0.32)e	0 (Ref.)	3.07 (0.36, 5.77)e
Diastolic blood pressure, mmHg	-3.08 (-3.99, -2.17)	<0.001	0 (Ref.)	0.50 (-2.33, 3.33)	-1.44 (-3.15, 0.28)	0 (Ref.)	3.21 (1.54, 4.88)e
Percentage weight regain (each 5%) during weight maintenance							
Fasting plasma glucose, mmol/l	0.13 (0.11, 0.15)	<0.001	0 (Ref.)	0.06 (-0.0004, 0.12)	0.05 (0.007, 0.10)e	0 (Ref.)	0.03 (-0.01, 0.07)
HbA_{1c}, mmol mol^{-1}	0.88 (0.81, 0.95)	<0.001	0 (Ref.)	0.25 (0.05, 0.45)e	0.16 (0.01, 0.32)e	0 (Ref.)	-0.04 (-0.18, 0.10)
Triacylglycerol, mmol/l	0.13 (0.11, 0.15)	<0.001	0 (Ref.)	-0.04 (-0.09, 0.01)	-0.04 (-0.08, 0.0006)	0 (Ref.)	-0.002 (-0.04, 0.03)
HDL-cholesterol, mmol/l	-0.04 (-0.04, -0.03)	<0.001	0 (Ref.)	-0.004 (-0.02, 0.01)	-0.009 (-0.02, 0.005)	0 (Ref.)	0.006 (-0.007, 0.02)
LDL-cholesterol, mmol/l	0.03 (0.008, 0.05)	0.007	0 (Ref.)	0.03 (-0.03, 0.10)	0.009 (-0.04, 0.06)	0 (Ref.)	-0.0004 (-0.05, 0.05)
Systolic blood pressure, mmHg	1.76 (1.29, 2.23)	<0.001	0 (Ref.)	0.54 (-0.79, 1.87)	1.41 (0.38, 2.44)e	0 (Ref.)	0.45 (-0.52, 1.42)
Diastolic blood pressure, mmHg	1.10 (0.81, 1.40)	<0.001	0 (Ref.)	0.07 (-0.76, 0.91)	0.62 (-0.03, 1.26)	0 (Ref.)	-0.07 (-0.68, 0.53)

Younger adults: 25−45 years; middle-aged adults: 46−54 years; older adults: 55−70 years. Analyses were performed using a linear mixed model including sex, age, ethnicity, baseline BMI, baseline smoking status, baseline alcohol drinking, baseline values of the outcome being considered, baseline energy intake and physical activity and percentage weight loss from baseline as fixed covariates and intervention centre as random effects. For the associations during weight maintenance, the models were additionally included intervention arm and percentage weight regain from 8 weeks. Percentage weight loss by age group or sex interaction terms were added, where appropriate.

[a]Values are adjusted mean changes (95% CI) in outcomes from baseline or from 8 weeks in all participants associated with each 8% of weight loss or 5% of weight regain.

[b]*p* for mean changes in all participants

[c]Reference group

[d]Values are relative mean changes (95% CI) in outcomes from baseline or from 8 weeks compared with the reference group associated with each 8% of weight loss or 5% of weight regain [e]Significant relative mean changes compared with the reference group, $p<0.05$

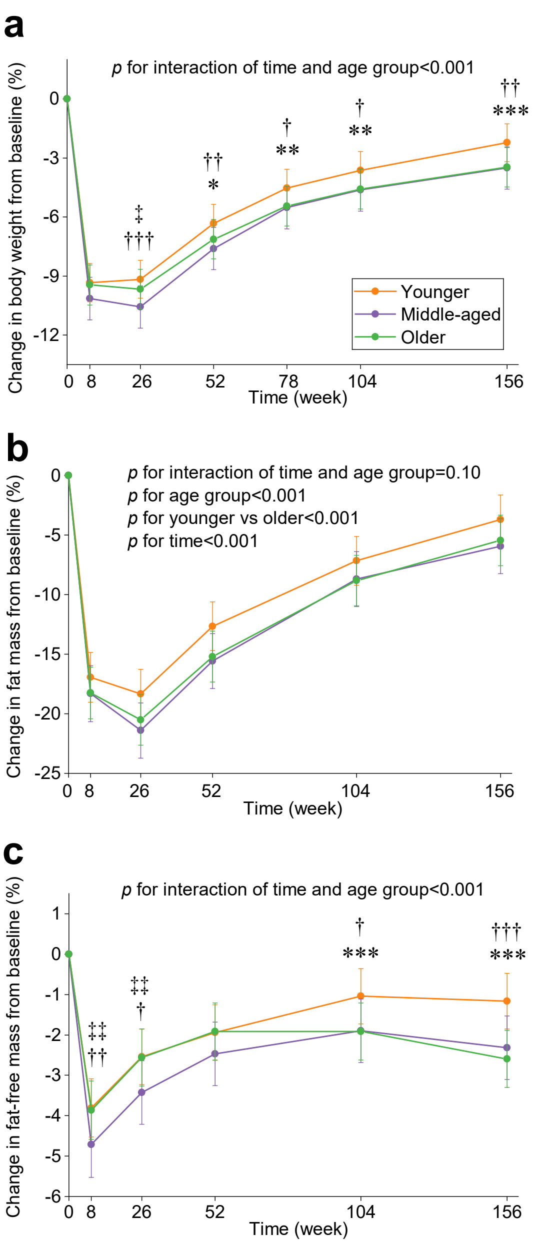

ESM Fig. 1 Changes in body weight and body composition by age group (n=2223). Values are estimated marginal mean (95% CI) in changes from baseline in percentage of body weight (**a**), percentage of fat mass (**b**), and percentage of fat-free mass (**c**). Younger adults: 25–45 years; middle-aged adults: 46–54 years; older adults: 55–70 years. Analyses were performed using a linear mixed model including sex, age, ethnicity, baseline BMI, baseline smoking status, baseline alcohol drinking, baseline values of the outcome being considered, baseline energy intake and physical activity, time-varying changes in energy intake and physical activity from baseline, intervention arm, time and interaction of time and age group as fixed covariates and participant identifier and intervention centre as random effects. Post hoc multiple comparisons with Bonferroni adjustment were performed to compare age groups at each time point, where appropriate. Older vs younger adults $^{*}p < 0.05$, $^{**}p < 0.01$, and $^{***}p < 0.001$; middle-aged vs younger adults $^{\dagger}p < 0.05$ and $^{\dagger\dagger}p < 0.01$; older vs middle-aged adults $^{\ddagger}p < 0.05$, $^{\ddagger\ddagger}p < 0.01$, and $^{\ddagger\ddagger\ddagger}p < 0.001$

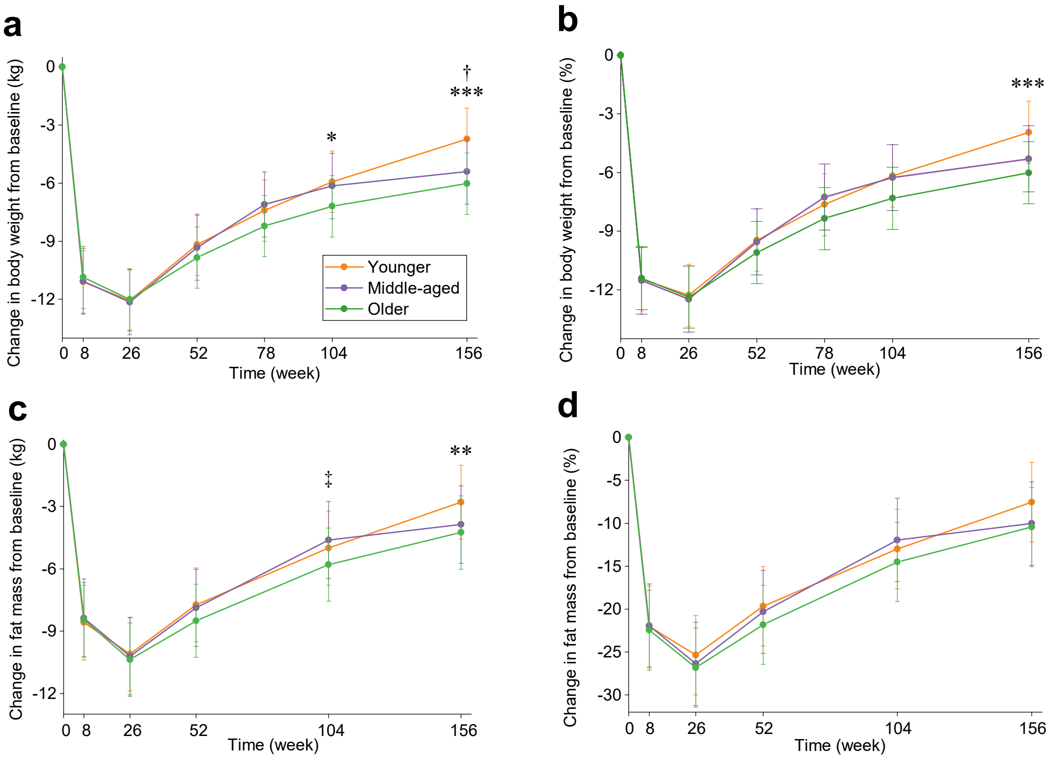

ESM Fig. 2 Changes in body weight and body composition by age group according to the complete-case analysis (n=962). Values are estimated marginal mean (95% CI) in changes from baseline in percentage of body weight (**a**), percentage of body weight (**b**), fat mass (**c**), and percentage of fat mass (**d**). Younger adults: 25−45 years; middle-aged adults: 46−54 years; older adults: 55−70 years. Analyses were performed using a linear mixed model including sex, age, ethnicity, baseline BMI, baseline smoking status, baseline alcohol drinking, baseline values of the outcome being considered, baseline energy intake and physical activity, time-varying changes in energy intake and physical activity from baseline, intervention arm, time and interaction of time and age group as fixed covariates and participant identifier and intervention centre as random effects. Post hoc multiple comparisons with Bonferroni adjustment were performed to compare age groups at each time point, where appropriate. Older vs younger adults $^{*}p < 0.05$, $^{**}p < 0.01$ and $^{***}p < 0.001$; middle-aged vs younger adults $^{\dagger}p < 0.05$; older vs middle-aged adults $^{\ddagger}p < 0.05$

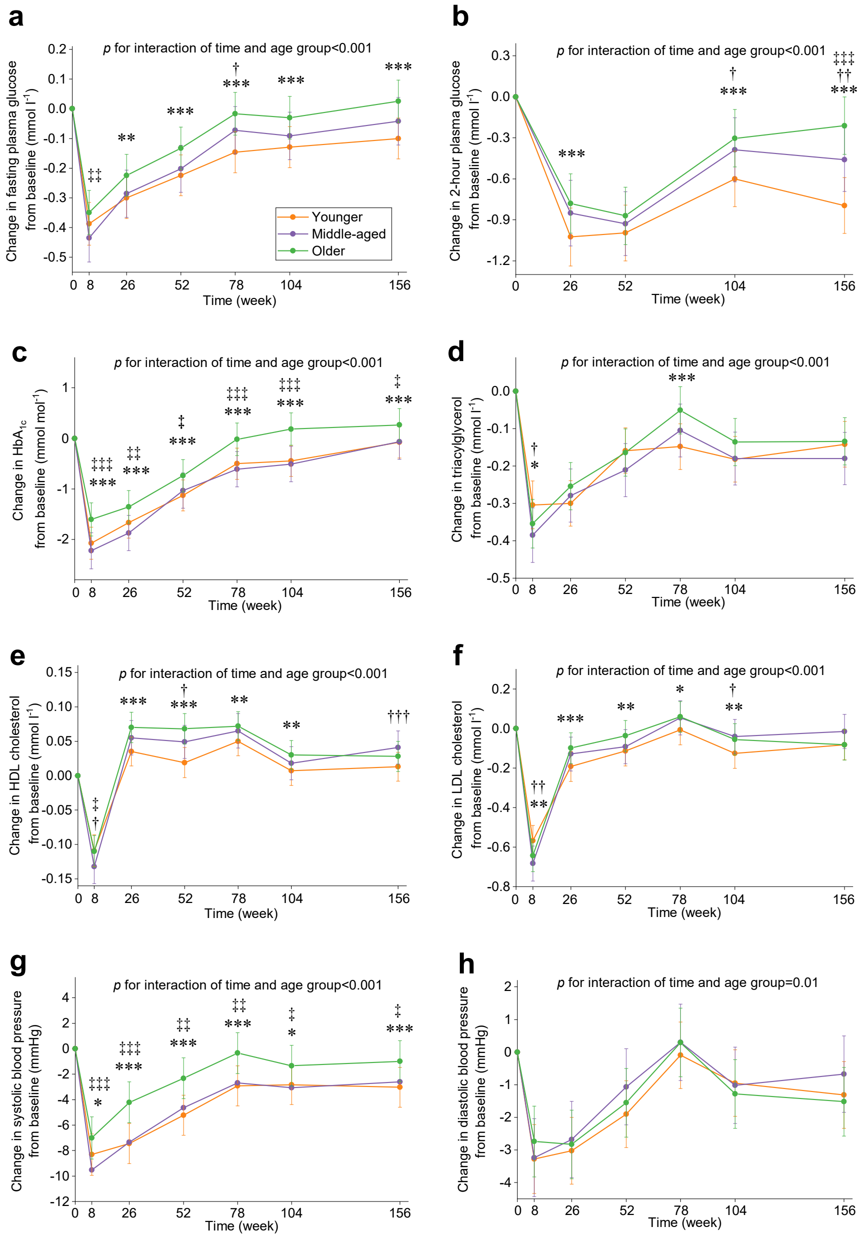

ESM Fig. 3 Weight loss-unadjusted changes in cardiometabolic health markers from baseline by age group (n=2223). Values are estimated marginal mean (95% CI) in changes from baseline in fasting plasma glucose (**a**), 2h plasma glucose (**b**), HbA_{1c} (**c**), triglycerides (**d**), HDL-cholesterol (**e**), LDL-cholesterol (**f**), systolic blood pressure (**g**), and diastolic blood pressure (**h**). Younger adults: 25−45 years; middle-aged adults: 46−54 years; older adults: 55−70 years. Analyses were performed using a linear mixed model including sex, age, ethnicity, baseline BMI, smoking status, alcohol drinking, baseline values of the outcome being considered, baseline energy intake and physical activity, time-varying changes in energy intake and physical activity from baseline, intervention arm, time and interaction of time and age group as covariates effects and participant identifier and intervention centre as random effects. Post hoc multiple comparisons with Bonferroni adjustment were performed to compare age groups at each time point. Older vs younger adults $^{*}p < 0.05$, $^{**}p < 0.01$ and $^{***}p < 0.001$; middle-aged vs younger adults $^{\dagger}p < 0.05$, $^{\dagger\dagger}p < 0.01$ and $^{\dagger\dagger\dagger}p < 0.001$; older vs middle-aged adults $^{\ddagger}p < 0.05$, $^{\ddagger\ddagger}p < 0.01$ and $^{\ddagger\ddagger\ddagger}p < 0.001$

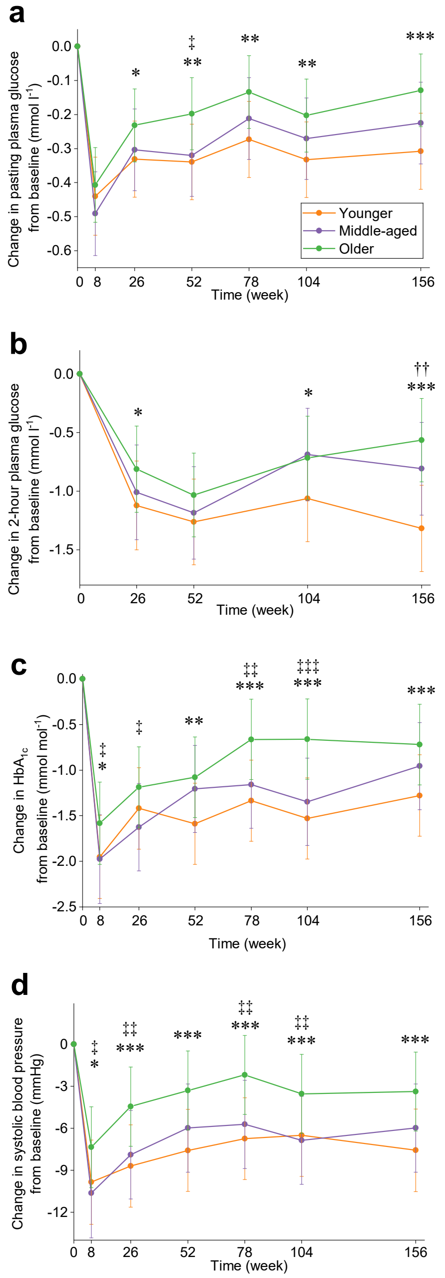

ESM Fig. 4 Weight loss-adjusted changes in cardiometabolic health markers from baseline by age group according to the complete-case analysis (n=962). Values are estimated marginal mean (95% CI) in changes from baseline in fasting plasma glucose (**a**), 2h plasma glucose (**b**), HbA_{1c} (**c**), and systolic blood pressure (**d**). Younger adults: 25−45 years; middle-aged adults: 46−54 years; older adults: 55−70 years. Analyses were performed using a linear mixed model including sex, age, ethnicity, baseline BMI, smoking status, alcohol drinking, baseline values of the outcome being considered, baseline energy intake and physical activity, time-varying changes in energy intake and physical activity from baseline, time-varying percentage weight loss from baseline, intervention arm, time and interaction of time and age group as covariates effects and participant identifier and intervention centre as random effects. Post hoc multiple comparisons with Bonferroni adjustment were performed to compare age groups at each time point. Older vs younger adults $^{*}p < 0.05$, $^{**}p < 0.01$ and $^{***}p < 0.001$; middle-aged vs younger adults $^{\dagger\dagger}p < 0.01$; older vs middle-aged adults $^{\ddagger}p < 0.05$, $^{\ddagger\ddagger}p < 0.01$ and $^{\ddagger\ddagger\ddagger}p < 0.001$

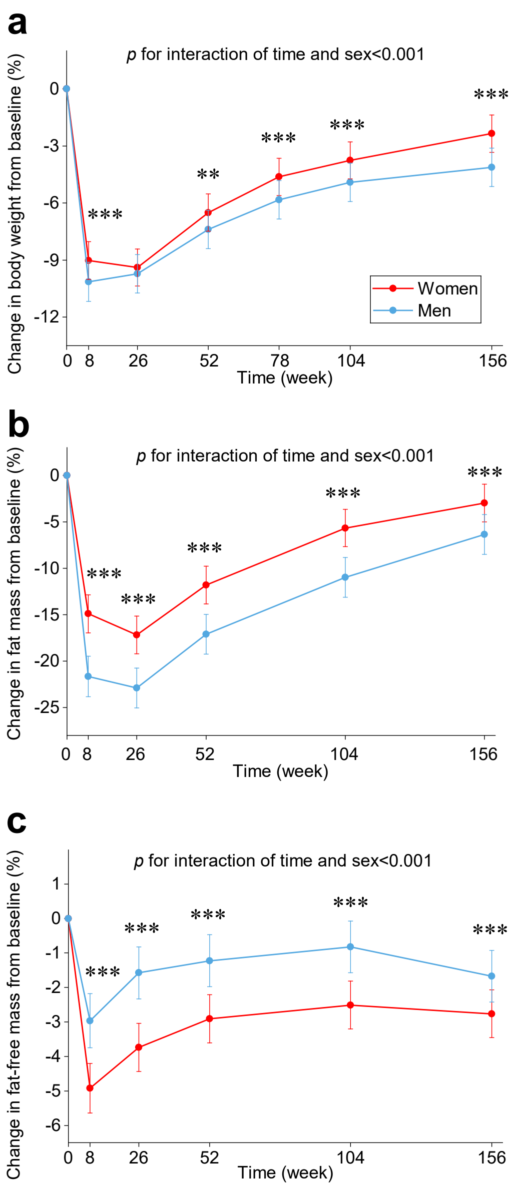

ESM Fig. 5 Changes in body weight and body composition from baseline in women and men (n=2223). Values are estimated marginal mean (95% CI) in changes from baseline in percentage of body weight (**a**), percentage of fat mass (**b**), and percentage of fat-free mass (**c**). Analyses were performed using a linear mixed model including sex, age, ethnicity, baseline BMI, baseline smoking status, baseline alcohol drinking, baseline values of the outcome being considered, baseline energy intake and physical activity, time-varying changes in energy intake and physical activity from baseline, intervention arm, time and interaction of time and sex as fixed covariates and participant identifier and intervention centre as random effects. Post hoc pairwise comparisons (independent samples *t* tests) were performed to compare women and men at each time point. Women vs men $^{**}p < 0.01$ and $^{***}p < 0.001$

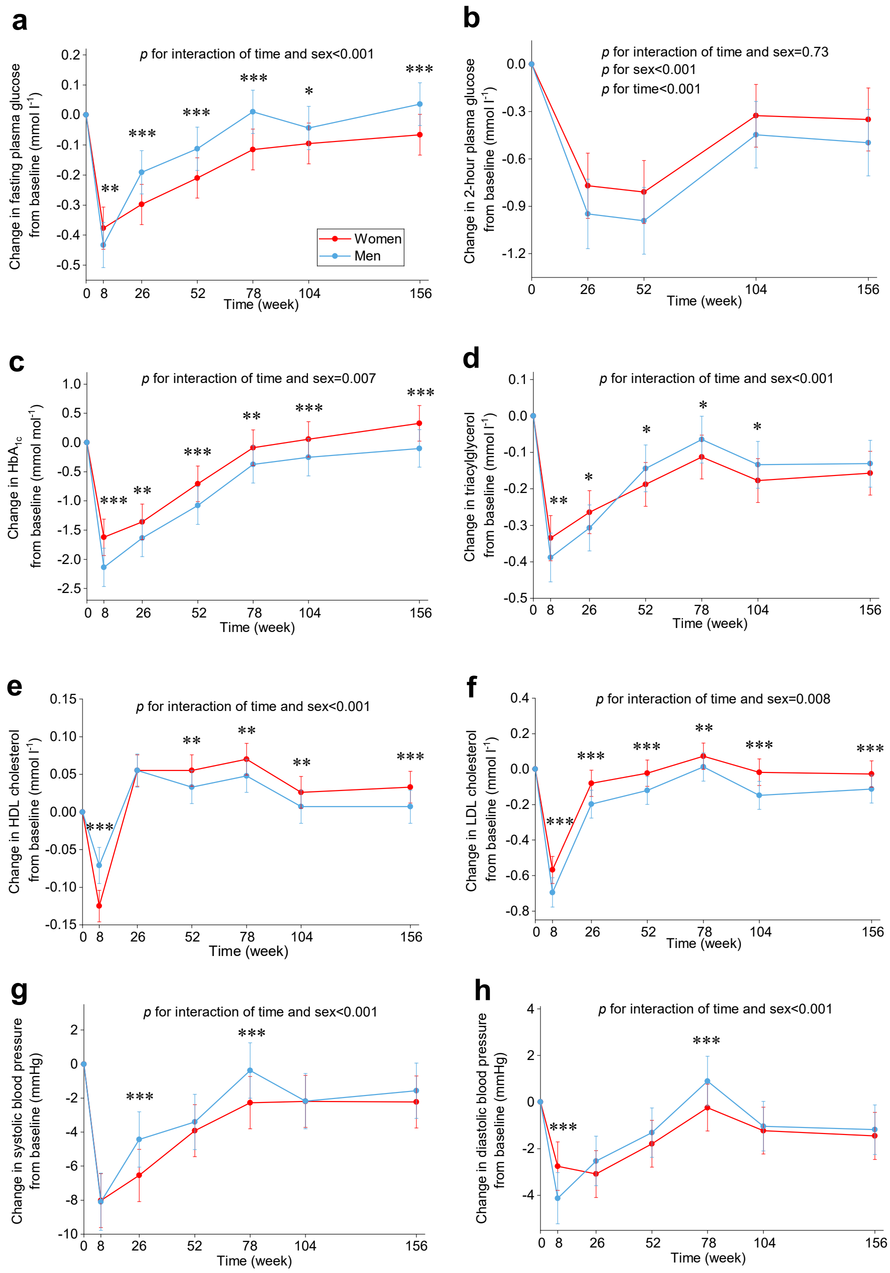

ESM Fig. 6 Weight loss-unadjusted changes in cardiometabolic health markers from baseline in women and men (n=2223). Values are estimated marginal mean (95% CI) in changes from baseline in fasting plasma glucose (**a**), 2h plasma glucose (**b**), HbA_{1c} (**c**), triglycerides (**d**), HDL cholesterol (**e**), LDL cholesterol (**f**), systolic blood pressure (**g**), and diastolic blood pressure (**h**). Analyses were performed using a linear mixed model including sex, age, ethnicity, baseline BMI, baseline smoking status, baseline alcohol drinking, baseline values of the outcome being considered, baseline energy intake and physical activity, time-varying changes in energy intake and physical activity from baseline, intervention arm, time and interaction of time and sex as fixed covariates and participant identifier and intervention centre as random effects. Post hoc pairwise comparisons (independent samples *t* tests) were performed to compare women and men at each timepoint, where appropriate. Women vs men $*p < 0.05$, $**p < 0.01$ and $***p < 0.001$

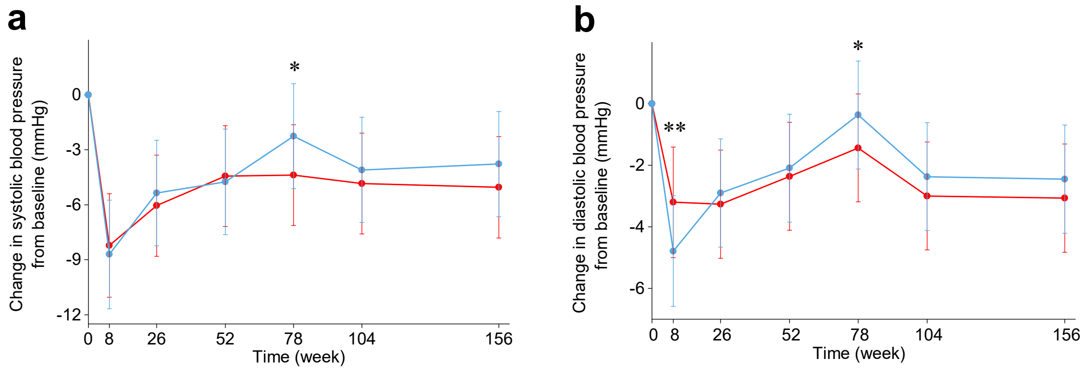

ESM Fig. 7 Weight loss-adjusted changes in cardiometabolic health markers from baseline in women and men according to the complete-case analysis (n=962). Values are estimated marginal mean (95% CI) in changes from baseline in systolic blood pressure (**a**) and diastolic blood pressure (**b**). Analyses were performed using a linear mixed model including sex, age, ethnicity, baseline BMI, baseline smoking status, baseline alcohol drinking, baseline values of the outcome being considered, baseline energy intake and physical activity, time-varying changes in energy intake and physical activity from baseline, time-varying percentage weight loss from baseline, intervention arm, time and interaction of time and sex as fixed covariates and participant identifier and intervention centre as random effects. Post hoc pairwise comparisons (independent samples *t* tests) were performed to compare women and men at each timepoint, where appropriate. Women vs men *$p < 0.05$, **$p < 0.01$ and ***$p < 0.001$
